# Supplementary figures and images for: The BABITT questionnaire for evaluation of bowel and bladder function in children who are introduced to assisted infant toilet training - content validity and feasibility
Source: PLoS One. 2025 Apr 30;20(4):e0320564. doi: 10.1371/journal.pone.0320564 (PMC12043185; doi:10.1371/journal.pone.0320564)

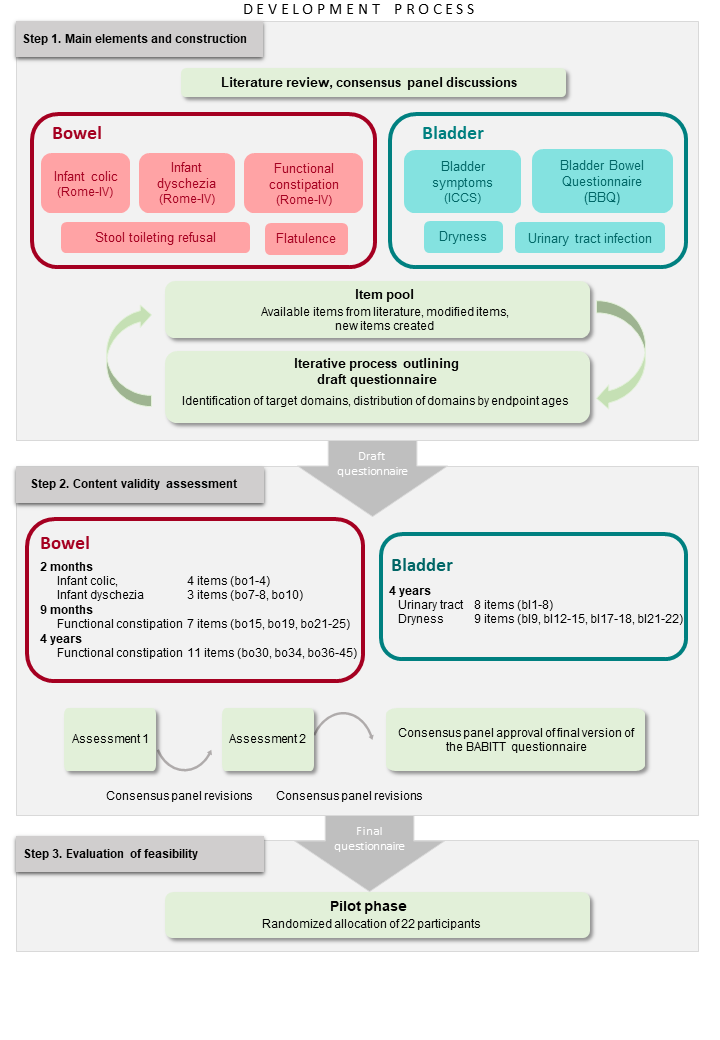

Supplement: S1 Fig — The three steps of the development process are graphically visualized. The construction process and main elements in Step 1. The CVI process in two assessment rounds and a final revision in Step 2. The evaluation of feasibility of the final questionnaire in the pilot phase in Step 3. (TIF) [file pone.0320564.s001.TIF]
