# Supplementary material for: The BABITT questionnaire for evaluation of bowel and bladder function in children who are introduced to assisted infant toilet training - content validity and feasibility
Source: PLoS One. 2025 Apr 30;20(4):e0320564. doi: 10.1371/journal.pone.0320564 (PMC12043185; doi:10.1371/journal.pone.0320564)
Supplement: S2 File — English translations of the BABITT questionnaires at 9 months, adding an indication of the content and structure that may aid the readership. As the translation from Swedish into English has not yet been linguistically and culturally validated, it should not be used for research purposes. (DOCX) [file pone.0320564.s002.docx]

# BABITT questionnaire at 9 months of age

*IN THE LAST MONTH*

1. Have there been major changes in your child’s daily life?
   1. No
   2. Yes, the arrival of a sibling
   3. Yes, change of residence
   4. Yes, separation
   5. Yes, illness in the family/close relatives
   6. Yes, death in the family/close relatives
   7. Yes, other

Comments (optional):______________________________________________________

1. Do you use a baby sling or baby carrier?
   1. Yes, daily
   2. Yes, 3-6 days a week
   3. Yes, 1-2 days a week
   4. Yes, but less than once a week
   5. No
2. Has your child’s development and growth been normal (according to assessments during visits to the Child Health Center)?
   1. Yes
   2. No

Comments (optional):______________________________________________________

1. Is your child allergic to cow’s milk protein (confirmed by healthcare professionals)?
   1. Yes
   2. No
   3. No, but my child has previously been allergic to cow’s milk protein
2. Is your child gluten intolerant (confirmed by healthcare professionals)?
   1. Yes
   2. No
3. Has your child suffered from constipation or other stomach issues that prompted you to seek medical care at any time?
   1. Yes
   2. No

Comments (optional):______________________________________________________

1. Has your child ever received treatment with antibiotics for urinary tract infection?
   1. Yes
   2. No

*IN THE LAST WEEK:*

1. How often do you breast feed your child (or give it breast milk in other ways)?
   1. Almost all meals
   2. Daily but not all meals
   3. A couple of times a week
   4. Once in a while
   5. Never
2. How often do you give your child formula?
   1. Almost all meals
   2. Daily but not all meals
   3. A couple of times a week
   4. Once in a while
   5. Never
3. How often do you give you child “regular” food (i.e. all foods that are not breast milk or formula)?
   1. Almost all meals
   2. Daily but not all meals
   3. A couple of times a week
   4. Once in a while
   5. Never
4. Does your child use diapers during daytime (at home)?
   1. Yes, my child uses diapers MOST OF THE TIME daytime (more than 75% of the time
   2. Yes, my child uses diapers SOMETIMES daytime (25‐75% of the time)
   3. Yes, my child uses diapers ONCE IN A WHILE (less than 25% of the time)
   4. No, my child NEVER uses diaper daytime

Comments (optional):______________________________________________________

1. What kind of diaper does your child mostly use (daytime)?
   1. Disposable diaper
   2. Cloth diaper
   3. Other
2. Try to estimate how many disposable diapers a day you used for your baby, last week.
   1. 0
   2. 1
   3. 2
   4. 3
   5. 4
   6. 5
   7. 6
   8. 7
   9. 8
   10. 9
   11. 10
   12. 11
   13. 12
   14. 13
   15. 14
   16. 15
   17. 16
   18. 17
   19. 18
   20. 19
   21. 20

Comments (optional):______________________________________________________

1. What kind of diaper does your child mostly use at night?
   1. Disposable diaper
   2. Cloth diaper
   3. Other
   4. My child doesn’t use diapers at night

*IN THE LAST MONTH:*

1. Does your child wet itself during the day (at home)?
   1. Yes, daily
   2. Yes, several times a week
   3. Yes, some times a month
   4. No my child does not wet itself
2. How large are the urinary leakages? (several options can be chosen(
   1. Underwear becomes moist
   2. Underwear and pants become wet
   3. Pants become wet down to knees/feet
3. How often was your child’s underwear stained or soiled with poop (at home)? (Periods of stomach flu excluded.)
   1. Yes, daily
   2. Yes, 3‐6 days a week
   3. Yes, 1‐2 days a week
   4. Yes, but less than once a week (i.e. 1‐3 the last month)
   5. Yes, but less than once a month
   6. No, my child never stains or soils its underwear

*IN THE LAST MONTH:*

1. Does your child pee at night? (several options can be chosen)
   1. Yes, my child is roused or picked up while sleeping to pee
   2. Yes, my child wakes up by itself to pee
   3. Yes, my child wets its bed
   4. Yes, my child wets its diaper
   5. No, my child does not pee at night
   6. Other

Comments (optional):______________________________________________________

1. In the morning, how often is your child’s bed or diaper wet?
   1. Every or every other night
   2. 2‐3 nights a week
   3. 3‐4 nights a month
   4. 1‐2 nights a month
   5. Less than 1 night a month
   6. Never
2. How soon after waking up does your child pee in the morning?
   1. Within 30 minutes
   2. Within 2 hours
   3. After 2 hours

*IN THE LAST MONTH:*

1. How often do you practice assisted infant toilet training with your child (successful output not required)?
   1. 5-7 days a week
   2. 3-4 days a week
   3. 1-2 days a week
   4. Less than 1 day a week (i.e. 1-3 times a month)
   5. Never
2. How many attempts are made, the days you practice infant toilet training?
   1. 8 times or more a day
   2. 4-7 times a day
   3. 1-3 times a day
3. Does your child have poops in a predictable pattern (e.g. after meals or sleep)?
   1. Yes, always (100 % of the time)
   2. Yes, most of the time (about 75 % of the time)
   3. Yes, sometimes (about 50 % of the time)
   4. Yes, once in a while (about 25 % of the time)
   5. No, never (0 % of the time)
4. Does your child pee in a predictable pattern (e.g. after meals or sleep)?
   1. Yes, always (100 % of the time)
   2. Yes, most of the time (about 75 % of the time)
   3. Yes, sometimes (about 50 % of the time)
   4. Yes, once in a while (about 25 % of the time)
   5. No, never (0 % of the time)
5. Does your child signal when it is time to poop?
   1. Yes, always (100 % of the time)
   2. Yes, most of the time (about 75 % of the time)
   3. Yes, sometimes (about 50 % of the time)
   4. Yes, once in a while (about 25 % of the time)
   5. No, never (0 % of the time)
6. Does your child signal when it is time to pee?
   1. Yes, always (100 % of the time)
   2. Yes, most of the time (about 75 % of the time)
   3. Yes, sometimes (about 50 % of the time)
   4. Yes, once in a while (about 25 % of the time)
   5. No, never (0 % of the time)
7. How does your child signal to pee or poop?
   1. Body language (e.g. whining, kicking legs)
   2. Searching for eye contact
   3. Specific sign (e.g. pointing at genitals/toilet or sign language)
   4. Verbally (specific word or sound)
   5. Moving towards potty/toilet
   6. Other

Comments (optional):______________________________________________________

1. Do you cue your child to pee with sound or procedure?
   1. Yes, always (100 % of the time)
   2. Yes, most of the time (about 75 % of the time)
   3. Yes, sometimes (about 50 % of the time)
   4. Yes, once in a while (about 25 % of the time)
   5. No, never (0 % of the time)

Comments (optional):______________________________________________________

1. Can your child hold the poop until you have arranged a receptacle?
   1. Yes, always (100 % of the time)
   2. Yes, most of the time (about 75 % of the time)
   3. Yes, sometimes (about 50 % of the time)
   4. Yes, once in a while (about 25 % of the time)
   5. No, never (0 % of the time)
2. Does your child hold the pee until you have arranged a receptacle?
   1. Yes, always (100 % of the time)
   2. Yes, most of the time (about 75 % of the time)
   3. Yes, sometimes (about 50 % of the time)
   4. Yes, once in a while (about 25 % of the time)
   5. No, never (0 % of the time)
3. Does your child ever protest when you attempt potty training?
   1. Yes, always (100 % of the time)
   2. Yes, most of the time (about 75 % of the time)
   3. Yes, sometimes (about 50 % of the time)
   4. Yes, once in a while (about 25 % of the time)
   5. No, never (0 % of the time)
4. How are the efforts to potty train distributed between you and your partner?
   1. I am the only one potty training
   2. Mostly me, but my partner makes some efforts
   3. We make equal efforts
   4. Mostly my partner, but I make some efforts
   5. My partner is the only one potty training
   6. Not applicable, I am a single parent

Comments (optional):______________________________________________________

*SINCE THE LAST SURVEY:*

1. How would you describe your experience of potty training your child?

*Mark the figure describing the situation.*

Very negative Very positive

1 2 3 4 5 6

1. Please describe your experiences and reflections on potty training your child. Consider both positive and negative aspects.

______________________________________________________________________________________________________________________________________________________________

1. Is your child being treated for constipation with bowel-regulating agents (i.e Laktulos®, Movicol®, Omnilax® or Forlax®) or enemas (i.e. Klyx®) to soften the stools?
   1. Yes, 3-7 days a week
   2. Yes, 1-2 days a week
   3. Yes, but less than once a week (i.e 1-3 times the last month)
   4. No, never
2. Have a doctor or nurse ever examined your child finding a large fecal mass in the rectum?
   1. Yes
   2. No
   3. Never examined

Comments (optional):______________________________________________________

*IN THE LAST MONTH:*

1. How often does your child poop?
   1. Several times a day
   2. Once a day or every other day
   3. 1-2 times a week or less often
2. Please estimate how many poops a day your child has
   1. 1 time a day
   2. 2 times a day
   3. 3 times a day
   4. 4 times a day
   5. 5 times a day
   6. 6 times a day
   7. 7 times a day
   8. 8 times a day
   9. 9 times a day
   10. 10 times a day
   11. 11 times a day
   12. More than 12 times a day
   13. I don’t know
3. Please estimate how many times a week your child poops
   1. 7 times a week
   2. 6 times a week
   3. 5 times a week
   4. 4 times a week
   5. 3 times a week
   6. I don’t know
4. Please estimate how many times a week your child poops
   1. 2 times a week
   2. Once a week
   3. Less than once a week
   4. I don’t know

Comments (optional):______________________________________________________

1. Does it ever hurt when your child poops?
   1. Yes, always (100 % of the time)
   2. Yes, most of the time (about 75 % of the time)
   3. Yes, sometimes (about 50 % of the time)
   4. Yes, once in a while (about 25 % of the time)
   5. No, never (0 % of the time)
2. Does your child ever have hard poops?
   1. Yes, always (100 % of the time)
   2. Yes, most of the time (about 75 % of the time)
   3. Yes, sometimes (about 50 % of the time)
   4. Yes, once in a while (about 25 % of the time)
   5. No, never (0 % of the time)
3. Does your child ever have unusually large poops (with a large diameter, unusually thick for the child’s age)?
   1. Yes, always (100 % of the time)
   2. Yes, most of the time (about 75 % of the time)
   3. Yes, sometimes (about 50 % of the time)
   4. Yes, once in a while (about 25 % of the time)
   5. No, never (0 % of the time)
4. Does your child ever actively postpone or hold in their poops?
   1. Yes, always (100 % of the time)
   2. Yes, most of the time (about 75 % of the time)
   3. Yes, sometimes (about 50 % of the time)
   4. Yes, once in a while (about 25 % of the time)
   5. No, never (0 % of the time)


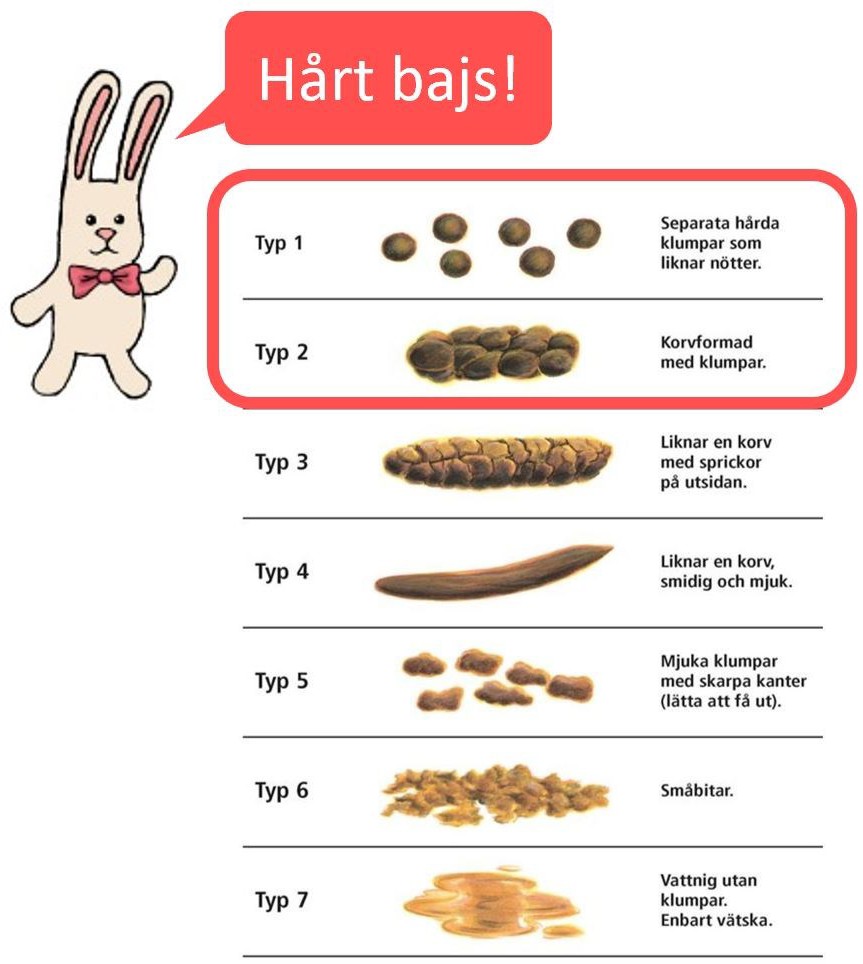


Hard poops!

1. Does your child strain and cry for more than 10 minutes when trying to poop?
   1. Yes, ongoing problems
   2. Yes, my child had previous problems
   3. No, never
2. How would you describe your child’s poops on these occasions?
   1. Hard
   2. Soft or mushy
   3. Stools are not passed
3. Does your child have problems with gas (flatulence)?
   1. Yes, 3-7 days a week
   2. Yes, 1-2 days a week
   3. Yes, but less than once a week (i.e. 1-3 times the last month)
   4. No, never
4. Does potty training seem to reduce dissatisfaction in your baby?
   1. Yes, always (100 % of the time)
   2. Yes, most of the time (about 75 % of the time)
   3. Yes, sometimes (about 50 % of the time)
   4. Yes, once in a while (about 25 % of the time)
   5. No, never (0 % of the time)
   6. Not applicable, my baby isn’t dissatisfied
