# Supplementary material for: The BABITT questionnaire for evaluation of bowel and bladder function in children who are introduced to assisted infant toilet training - content validity and feasibility
Source: PLoS One. 2025 Apr 30;20(4):e0320564. doi: 10.1371/journal.pone.0320564 (PMC12043185; doi:10.1371/journal.pone.0320564)
Supplement: S3 File — English translations of the BABITT questionnaires at 2.5 years, adding an indication of the content and structure that may aid the readership. As the translation from Swedish into English has not yet been linguistically and culturally validated, it should not be used for research purposes. (DOCX) [file pone.0320564.s003.docx]

# BABITT questionnaire at 2.5 years of age

*SINCE THE LAST SURVEY*

1. Have there been major changes in your child’s daily life?
   1. No
   2. Yes, the arrival of a sibling
   3. Yes, change of residence
   4. Yes, separation
   5. Yes, illness in the family/close relatives
   6. Yes, death in the family/close relatives
   7. Yes, other

Comments (optional):______________________________________________________

1. Are you living with your child’s other parent/legal guardian?
   1. Yes, I am married/cohabiting
   2. I am separated with joint custody of my child.
   3. No, I am separated with sole custody of my child
   4. Other

Comments (optional):______________________________________________________

1. To what extent does your child live with you?
   1. More than 75% of the time
   2. 25-75 % of the time
   3. Less than 25% of the time
2. Are there other children (e.g. full/half or step-siblings) in the household?
   1. Yes
   2. No
3. Specify the ages of the other children (e.g. full/half or step-siblings) and indicate whether they live full-time or part-time in the household:

The sibling lives in the household:

Age (years):_____

- >75% of the time
- 25-75% of the time
- >25% of the time

*(Automatic display of as many options as needed in web-survey)*

Comments (optional):______________________________________________________

1. Has your child’s development and growth been normal (according to assessments during visits to the Child Health Center)?
   1. Yes
   2. No

Comments (optional):______________________________________________________

1. Has your child suffered from constipation or other stomach issues that prompted you to seek medical care at any time?
   1. Yes
   2. No

Comments (optional):______________________________________________________

1. Has your child ever received treatment with antibiotics for urinary tract infection?
   1. Yes
   2. No

*IN THE LAST MONTH:*

1. Does your child attend preschool/daycare?
   1. Yes
   2. No
   3. Other
2. How many hours a week does your child spend in preschool/daycare, on average?
   1. 20 hours a week or less
   2. 21-39 hours a week
   3. 40 hours a week or more
3. Does your child use the potty/toilet to pee at preschool/daycare?
   1. Yes, often
   2. Yes, sometimes
   3. No, my child always pees in a diaper
   4. No, my child refrains from peeing
4. Does your child use the potty/toilet to poop at preschool/daycare?
   1. Yes, often
   2. Yes, sometimes
   3. No, my child always poops in a diaper
   4. No, my child refrains from having poops

How accurate are the following statements?

*Mark the figure describing the situation*

1. My child received sufficient support in toilet training from preschool/daycare.

Completely disagree Completely agree

1 2 3 4 5 6

1. Preschool/daycare accommodates regular toilet routines for my child.

Completely disagree Completely agree

1 2 3 4 5 6

1. Please describe your experiences and reflections on toilet training and toilet routines at preschool/daycare. Consider both positive and negative aspects.

______________________________________________________________________________________________________________________________________________________________

1. Does your child use diapers during daytime (at home)?
   1. Yes, my child uses diapers MOST OF THE TIME daytime (more than 75% of the time
   2. Yes, my child uses diapers SOMETIMES daytime (25‐75% of the time)
   3. Yes, my child uses diapers ONCE IN A WHILE (less than 25% of the time)
   4. No, my child NEVER uses diaper daytime

Comments (optional):______________________________________________________

1. What kind of diaper does your child mostly use (daytime)?
   1. Disposable diaper
   2. Cloth diaper
   3. Other
2. What kind of diaper does your child mostly use at night?
   1. Disposable diaper
   2. Cloth diaper
   3. Other
   4. My child doesn’t use diapers at night

*IN THE LAST MONTH:*

1. Does your child wet itself during the day (at home)?
   1. Yes, daily
   2. Yes, several times a week
   3. Yes, some times a month
   4. No, my child does not wet itself
2. How large are the urinary leakages? (Several options can be chosen.(
   1. Underwear becomes moist
   2. Underwear and pants become wet
   3. Pants become wet down to knees/feet
3. How often was your child’s underwear stained or soiled with poop (at home)? (Periods of stomach flu excluded.)
   1. Yes, daily
   2. Yes, 3‐6 days a week
   3. Yes, 1‐2 days a week
   4. Yes, but less than once a week (i.e. 1‐3 the last month)
   5. Yes, but less than once a month
   6. No, my child never stains or soils its underwear

*IN THE LAST MONTH:*

1. Does your child pee at night? (Several options can be chosen.)
   1. Yes, my child is roused or picked up while sleeping to pee
   2. Yes, my child wakes up by itself to pee
   3. Yes, my child wets its bed
   4. Yes, my child wets its diaper
   5. No, my child does not pee at night
   6. Other

Comments (optional):______________________________________________________

1. In the morning, how often is your child’s bed or diaper wet?
   1. Every or every other night
   2. 2‐3 nights a week
   3. 3‐4 nights a month
   4. 1‐2 nights a month
   5. Less than 1 night a month
   6. Never
2. How soon after waking up does your child pee in the morning?
   1. Within 30 minutes
   2. Within 2 hours
   3. After 2 hours

*IN THE LAST MONTH:*

1. How often does your child sit on the potty/toilet (successful output not required)?
   1. 5-7 days a week
   2. 3-4 days a week
   3. 1-2 days a week
   4. Less than 1 day a week (i.e. 1-3 times a month)
   5. Never
2. How many times per day does your child sit on the potty/toilet (successful output not required)?
   1. 8 times or more a day
   2. 4-7 times a day
   3. 1-3 times a day
3. Does your child have poops in a predictable pattern (e.g. after meals or sleep)?
   1. Yes, always (100 % of the time)
   2. Yes, most of the time (about 75 % of the time)
   3. Yes, sometimes (about 50 % of the time)
   4. Yes, once in a while (about 25 % of the time)
   5. No, never (0 % of the time)
4. Does your child pee in a predictable pattern (e.g. after meals or sleep)?
   1. Yes, always (100 % of the time)
   2. Yes, most of the time (about 75 % of the time)
   3. Yes, sometimes (about 50 % of the time)
   4. Yes, once in a while (about 25 % of the time)
   5. No, never (0 % of the time)
5. Does your child signal when it is time to poop?
   1. Yes, always (100 % of the time)
   2. Yes, most of the time (about 75 % of the time)
   3. Yes, sometimes (about 50 % of the time)
   4. Yes, once in a while (about 25 % of the time)
   5. No, never (0 % of the time)
6. Does your child signal when it is time to pee?
   1. Yes, always (100 % of the time)
   2. Yes, most of the time (about 75 % of the time)
   3. Yes, sometimes (about 50 % of the time)
   4. Yes, once in a while (about 25 % of the time)
   5. No, never (0 % of the time)
7. Can your child hold the poop until you have arranged a receptacle?
   1. Yes, always (100 % of the time)
   2. Yes, most of the time (about 75 % of the time)
   3. Yes, sometimes (about 50 % of the time)
   4. Yes, once in a while (about 25 % of the time)
   5. No, never (0 % of the time)
8. Does your child hold the pee until you have arranged a receptacle?
   1. Yes, always (100 % of the time)
   2. Yes, most of the time (about 75 % of the time)
   3. Yes, sometimes (about 50 % of the time)
   4. Yes, once in a while (about 25 % of the time)
   5. No, never (0 % of the time)
9. How are/were the efforts to toilet train distributed between you and your partner?
   1. I am/was the only one toilet training
   2. Mostly I, but my partner makes/made some efforts
   3. We make/made equal efforts
   4. Mostly my partner, but I make/made some efforts
   5. My partner is/was the only one toilet training
   6. Not applicable, I am a single parent

Comments (optional):______________________________________________________

*SINCE THE LAST SURVEY:*

1. How would you describe your experience of toilet training your child?

*Mark the figure describing the situation.*

Very negative Very positive

1 2 3 4 5 6

1. Please describe your experiences and reflections on toilet training your child. Consider both positive and negative aspects.

______________________________________________________________________________________________________________________________________________________________

*IN THE LAST MONTH:*

1. How often does your child pee during the day?
   1. 8 times or more a day
   2. 4‐7 times a day
   3. 3 times a day
   4. 1‐2 times a day
2. Does your child ever rush to the toilet (sudden, urgent need) without preceding signs of needing to pee?
   1. Yes, daily
   2. Yes, several times a week
   3. Yes, a few times a month
   4. No, never
3. Does your child ever postpone a voiding despite needing to pee?
   1. Yes, daily
   2. Yes, several times a week
   3. Yes, a few times a month
   4. No, never
4. Does your child ever react to the urge to pee by pressing hands to its genitals, sitting on its heels or resisting the urge in other ways?
   1. Yes, daily
   2. Yes, several times a week
   3. Yes, a few times a month
   4. No, never
5. Does your child ever strain when voiding?
   1. Yes, daily
   2. Yes, several times a week
   3. Yes, a few times a month
   4. No, never
6. When your child is about to pee, is the voiding ever hesitant to start with?
   1. Yes, daily
   2. Yes, several times a week
   3. Yes, a few times a month
   4. No, never
7. Does your child ever have intermittent flow when voiding (several starts and stops when peeing)?
   1. Yes, daily
   2. Yes, several times a week
   3. Yes, a few times a month
   4. No, never

How accurate are the following statements?

*Mark the figure describing the situation.*

1. My child takes the initiative to visit the toilet (at home)

Completely agree Completely disagree

1 2 3 4 5 6

1. It is important for an adult to keep track of my child’s toilet habits.

Completely agree Completely disagree

1 2 3 4 5 6

1. Is your child being treated for constipation with bowel regulating agents (i.e Laktulos®, Movicol®, Omnilax® or Forlax®) or enemas (i.e. Klyx®) to soften the stools?
   1. Yes, 3-7 days a week
   2. Yes, 1-2 days a week
   3. Yes but less than once a week (i.e 1-3 times the last month)
   4. No, never
2. Did a doctor or nurse ever examine your child finding a large fecal mass in the rectum?
   1. Yes
   2. No
   3. Never examined

Comments (optional):______________________________________________________

*IN THE LAST MONTH:*

1. How often does your child poop?
   1. Once or several times a day
   2. Every other day
   3. 1-2 times a week
   4. Less than once a week
2. Please estimate how many poops a day your child has
   1. 1 time a day
   2. 2 times a day
   3. 3 times a day
   4. 4 times a day
   5. 5 times a day
   6. 6 times a day
   7. 7 times a day
   8. 8 times a day
   9. 9 times a day
   10. 10 times a day
   11. 11 times a day
   12. More than 12 times a day
   13. I don’t know
3. Please estimate how many times a week your child poops.
   1. 7 times a week
   2. 6 times a week
   3. 5 times a week
   4. 4 times a week
   5. 3 times a week
   6. I don’t know
4. Please estimate how many times a week your child poops.
   1. 2 times a week
   2. Once a week
   3. Less than once a week
   4. I don’t know

Comments (optional):______________________________________________________

1. Does it ever hurt when your child poops?
   1. Yes, always (100 % of the time)
   2. Yes, most of the time (about 75 % of the time)
   3. Yes, sometimes (about 50 % of the time)
   4. Yes, once in a while (about 25 % of the time)
   5. No, never (0 % of the time)
2. Does your child ever have hard poops?
   1. Yes, always (100 % of the time)
   2. Yes, most of the time (about 75 % of the time)
   3. Yes, sometimes (about 50 % of the time)
   4. Yes, once in a while (about 25 % of the time)
   5. No, never (0 % of the time)
3. Does your child ever have unusually large poops (with a large diameter, unusually thick for the child’s age)?
   1. Yes, always (100 % of the time)
   2. Yes, most of the time (about 75 % of the time)
   3. Yes, sometimes (about 50 % of the time)
   4. Yes, once in a while (about 25 % of the time)
   5. No, never (0 % of the time)
4. Does your child ever actively postpone or hold in their poops?
   1. Yes, always (100 % of the time)
   2. Yes, most of the time (about 75 % of the time)
   3. Yes, sometimes (about 50 % of the time)
   4. Yes, once in a while (about 25 % of the time)
   5. No, never (0 % of the time)
5. Does your child ever have poops so big that it clogs the toilet?
   1. Yes, always (100 % of the time)
   2. Yes, most of the time (about 75 % of the time)
   3. Yes, sometimes (about 50 % of the time)
   4. Yes, once in a while (about 25 % of the time)
   5. No, never (0 % of the time)
6. Does your child display some kind of procedure when pooping, i.e. holding on to a chair, tiptoeing, standing straight with tense legs or rocking back and forth?
   1. Yes, always (100 % of the time)
   2. Yes, most of the time (about 75 % of the time)
   3. Yes, sometimes (about 50 % of the time)
   4. Yes, once in a while (about 25 % of the time)
   5. No, never (0 % of the time)

If option a-d, please elaborate: ____________________________________________________________________________________________________________________________________________________________________


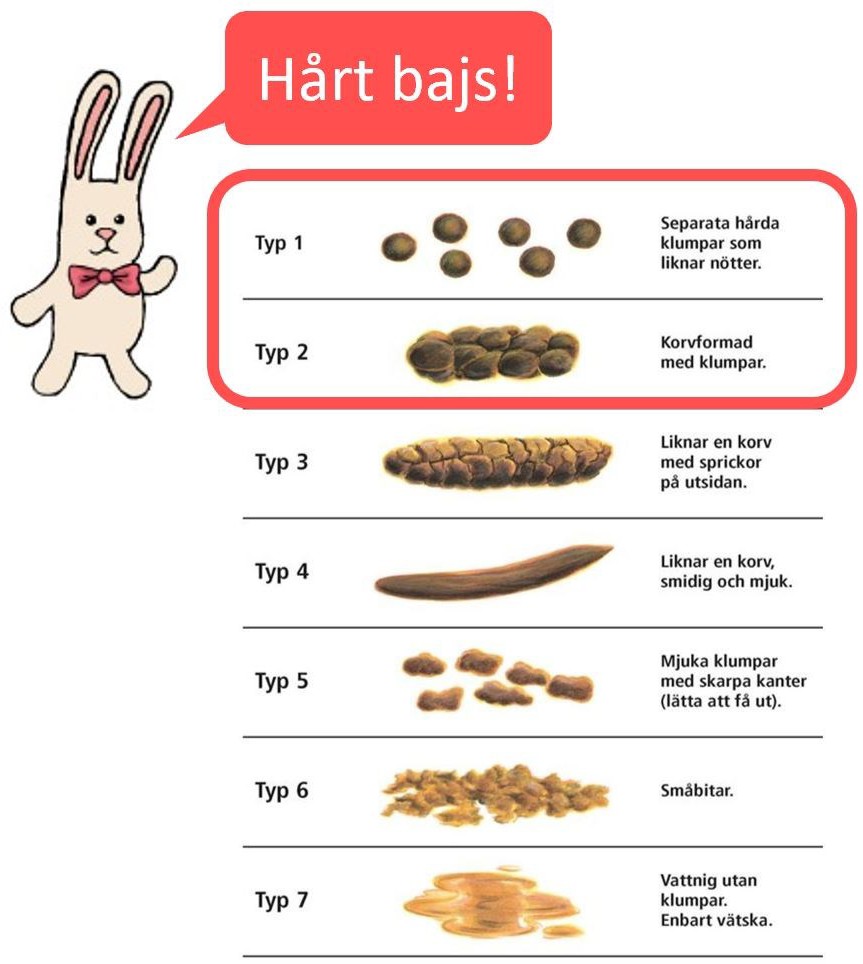


Hard poops!

1. Did your child ever go through a phase of consistently avoiding using the potty/toilet to poop (despite being comfortable peeing there), when it asked instead for a diaper in which to poop.?
   1. Yes, for more than 1 month
   2. Yes, for less than 1 month
   3. No
2. How old was your child when this occurred? *Several options can be chosen*
   1. 0-1 year (0-11 months)
   2. 1-2 years (12-23 months)
   3. 2-3 years (24-35 months)
   4. 3-4 years (36-47 months)
   5. Ongoing

Comments (optional):______________________________________________________
