# Supplementary material for: The BABITT questionnaire for evaluation of bowel and bladder function in children who are introduced to assisted infant toilet training - content validity and feasibility
Source: PLoS One. 2025 Apr 30;20(4):e0320564. doi: 10.1371/journal.pone.0320564 (PMC12043185; doi:10.1371/journal.pone.0320564)
Supplement: S4 File — English translations of the BABITT questionnaires at 4 years, adding an indication of the content and structure that may aid the readership. As the translation from Swedish into English has not yet been linguistically and culturally validated, it should not be used for research purposes. (DOCX) [file pone.0320564.s004.docx]

# BABITT questionnaire at 4 years of age

1. 1. Does your child use diapers during daytime (at home)?
   1. Yes, my child uses diapers MOST OF THE TIME daytime (more than 75% of the time)
   2. Yes, my child uses diapers SOMETIMES daytime (25‐75% of the time)
   3. Yes, my child uses diapers ONCE IN A WHILE daytime (less than 25% of the time)
   4. No, my child NEVER uses diaper daytime

Comments (optional):______________________________________________________

*IN THE LAST THREE MONTHS:*

1. Does your child wet itself during the day (at home)?
   1. Yes, daily
   2. Yes, several times a week
   3. Yes, some times a month
   4. No, my child does not wet itself
2. How large are the urinary leakages? (several options can be chosen(
   1. Underwear becomes moist
   2. Underwear and pants become wet
   3. Pants become wet down to knees/feet
3. How long has the urinary leakage occurred to this extent?
   1. Less than 1 month
   2. 1‐3 months
   3. More than 3 months

*IN THE LAST MONTH:*

1. How often was your child’s underwear stained or soiled with poop (at home)? (Periods of stomach flu excluded)
   1. Yes, daily
   2. Yes, 3‐6 days a week
   3. Yes, 1‐2 days a week
   4. Yes, but less than 1 day a week (i.e. 1‐3 the last month)
   5. Yes, but less than 1 day a month
   6. No, my child never soils its underwear

*IN THE LAST THREE MONTHS:*

1. Does your child pee at night? (Several options can be chosen.)
   1. Yes, my child is roused or picked up while sleeping to pee
   2. Yes, my child wakes up by itself to pee
   3. Yes, my child wets its bed
   4. Yes, my child wets its diaper
   5. No, my child does not pee at night
   6. Other

Comments (optional):______________________________________________________

1. How often is your child’s bed or diaper wet in the morning?
   1. Every or every other night
   2. 2‐3 nights a week
   3. 3‐4 nights a month
   4. 1‐2 nights a month
   5. Less than 1 night a month
   6. Never
2. At what age did your child become dry at night? (If your child needs to pee at night it wakes up by itself)
   1. Less than 1 year old (0-11 months)
   2. 1‐1.5 years (12‐18 months)
   3. 1.5‐2 years (19‐23 months)
   4. 2‐2.5 years (24‐29 months)
   5. 2.5‐3 years )30‐35 months)
   6. 3 -3.5 years (36‐41 months)
   7. 3.5‐4 years (42‐47 months)
3. How soon after waking up does your child pee in the morning?
   1. Within 30 minutes
   2. Within 2 hours
   3. After 2 hours
4. At what age did your child become dry/diaper free daytime?
   1. Less than 1 year old (0-11 months)
   2. 1‐1.5 years (12‐18 months)
   3. 1.5‐2 years (19‐23 months)
   4. 2‐2.5 years (24‐29 months)
   5. 2.5‐3 years )30‐35 months)
   6. 3‐3.5 years (36‐41 months)
   7. 3.5‐4 years (42‐47 months)
   8. Toilet training still not finished
   9. I don’t know
5. At what age did you initiate toilet training (daily attempts on potty/toilet)?
   1. Less than 1 year (0‐11 months)
   2. 1‐1.5 years (12‐18 months)
   3. 1.5‐2 years (19‐23 months)
   4. 2‐2.5 years (24‐29 months)
   5. 2.5‐3 years (30‐35 months)
   6. 3‐3.5 years (36‐41 months)
   7. 3.5‐4 years (42‐47 months)
   8. Not started yet
   9. I don’t know
6. More precisely, how old was your infant when your started daily toilet training, or so-called elimination communication?
   1. 0-3 months
   2. 0‐3 months
   3. 4‐5 months
   4. 6‐9 months
   5. 10-11 months

Comments (optional):______________________________________________________

1. How are/were the efforts to toilet train distributed between you and your partner?
   1. I am/was the only one toilet training
   2. Mostly me, but my partner makes/made some efforts
   3. We make/made equal efforts
   4. Mostly my partner, but I make/made some efforts
   5. My partner is/was the only one toilet training
   6. Not applicable, I am a single parent

Comments (optional):______________________________________________________

*SINCE THE LAST SURVEY:*

1. How would you describe your experience of toilet training with your child?

*Mark the figure describing your experience.*

Very negative Very positive

1 2 3 4 5 6

1. Please describe your experiences and reflections on toilet training with your child. Consider both positive and negative aspects.

______________________________________________________________________________________________________________________________________________________________________________________________________________________________________________________

*IN THE LAST MONTH:*

1. How often does your child pee during the day?
   1. 8 times or more a day
   2. 4‐7 times a day
   3. 3 times a day
   4. 1‐2 times a day
2. Does your child ever rush to the toilet (sudden, urgent need) without preceding signs of needing to pee?
   1. Yes, daily
   2. Yes, several times a week
   3. Yes, a few times a month
   4. No, never
3. Does your child ever postpone a voiding despite needing to pee?
   1. Yes, daily
   2. Yes, several times a week
   3. Yes, a few times a month
   4. No, never
4. Does your child ever react to the urge to pee by pressing hands to its genitals, sitting on its heels or resisting the urge in other ways?
   1. Yes, daily
   2. Yes, several times a week
   3. Yes, a few times a month
   4. No, never
5. Does your child ever strain when voiding?
   1. Yes, daily
   2. Yes, several times a week
   3. Yes, a few times a month
   4. No, never
6. When your child is about to pee, is the voiding ever hesitant to start with?
   1. Yes, daily
   2. Yes, several times a week
   3. Yes, a few times a month
   4. No, never
7. Does your child ever have intermittent flow when voiding (several starts and stops when peeing)?
   1. Yes, daily
   2. Yes, several times a week
   3. Yes, a few times a month
   4. No, never

How accurate are the following statements?

*Mark the figure describing the situation.*

1. My child takes the initiative to visit the toilet (at home)

Completely agree Completely disagree

1 2 3 4 5 6

1. It is important for an adult to keep track of my child’s toilet habits.

Completely agree Completely disagree

1 2 3 4 5 6

1. Is your child being treated for constipation with bowel regulating agents (i.e Laktulos®, Movicol®, Omnilax® or Forlax®) or enemas (i.e. Klyx®) to soften the stools?
   1. Yes, at present (for more than 1 year)
   2. Yes, at present (for less than 1 year)
   3. Yes, during previous episodes
   4. No, my child has never had treatment

*IN THE LAST MONTH:*

1. Did a doctor or nurse ever examine your child finding a large fecal mass in the rectum?
   1. Yes
   2. No
   3. Never examined

Comments (optional):______________________________________________________

1. How often does your child poop?
   1. Once or several times a day
   2. Every other day
   3. 1-2 times a week
   4. Less than once a week
2. Please estimate how many poops a day your child has.
   1. 1 time a day
   2. 2 times a day
   3. 3 times a day
   4. 4 times a day
   5. 5 times a day
   6. 6 times a day
   7. 7 times a day
   8. 8 times a day
   9. 9 times a day
   10. 10 times a day
   11. 11 times a day
   12. More than 12 times a day
   13. I don’t know
3. Please estimate how many times a week you child poops
   1. 7 times a week
   2. 6 times a week
   3. 5 times a week
   4. 4 times a week
   5. 3 times a week
   6. I don’t know
4. Please estimate how many times a week your child poops
   1. 2 times a week
   2. Once a week
   3. I don’t know

*If no option is applicable, please comment: ____________________________________________________________________________________________________________________________________________________________________*

1. Does it ever hurt when your child has poops?
   1. Yes, always (100 % of the time)
   2. Yes, most of the time (about 75 % of the time)
   3. Yes, sometimes (about 50 % of the time)
   4. Yes, once in a while (about 25 % of the time)
   5. No, never (0 % of the time)
2. Does your child ever have hard poops?
   1. Yes, always (100 % of the time)
   2. Yes, most of the time (about 75 % of the time)
   3. Yes, sometimes (about 50 % of the time)
   4. Yes, once in a while (about 25 % of the time)
   5. No, never (0 % of the time)
3. Does your child ever have unusually large poops (with a large diameter, unusually thick for the child’s age)?
   1. Yes, always (100 % of the time)
   2. Yes, most of the time (about 75 % of the time)
   3. Yes, sometimes (about 50 % of the time)
   4. Yes, once in a while (about 25 % of the time)
   5. No, never (0 % of the time)
4. Does your child ever actively postpone or hold in their poops?
   1. Yes, always (100 % of the time)
   2. Yes, most of the time (about 75 % of the time)
   3. Yes, sometimes (about 50 % of the time)
   4. Yes, once in a while (about 25 % of the time)
   5. No, never (0 % of the time)
5. Does your child ever have poops so big that it clogs the toilet?
   1. Yes, always (100 % of the time)
   2. Yes, most of the time (about 75 % of the time)
   3. Yes, sometimes (about 50 % of the time)
   4. Yes, once in a while (about 25 % of the time)
   5. No, never (0 % of the time)
6. Does your child display some kind of procedure when having poops, i.e. holding on to a chair, tiptoeing, standing straight with tense legs or rocking back and forth?
   1. Yes, always (100 % of the time)
   2. Yes, most of the time (about 75 % of the time)
   3. Yes, sometimes (about 50 % of the time)
   4. Yes, once in a while (about 25 % of the time)
   5. No, never (0 % of the time)

If option a-d, please elaborate: ____________________________________________________________________________________________________________________________________________________________________


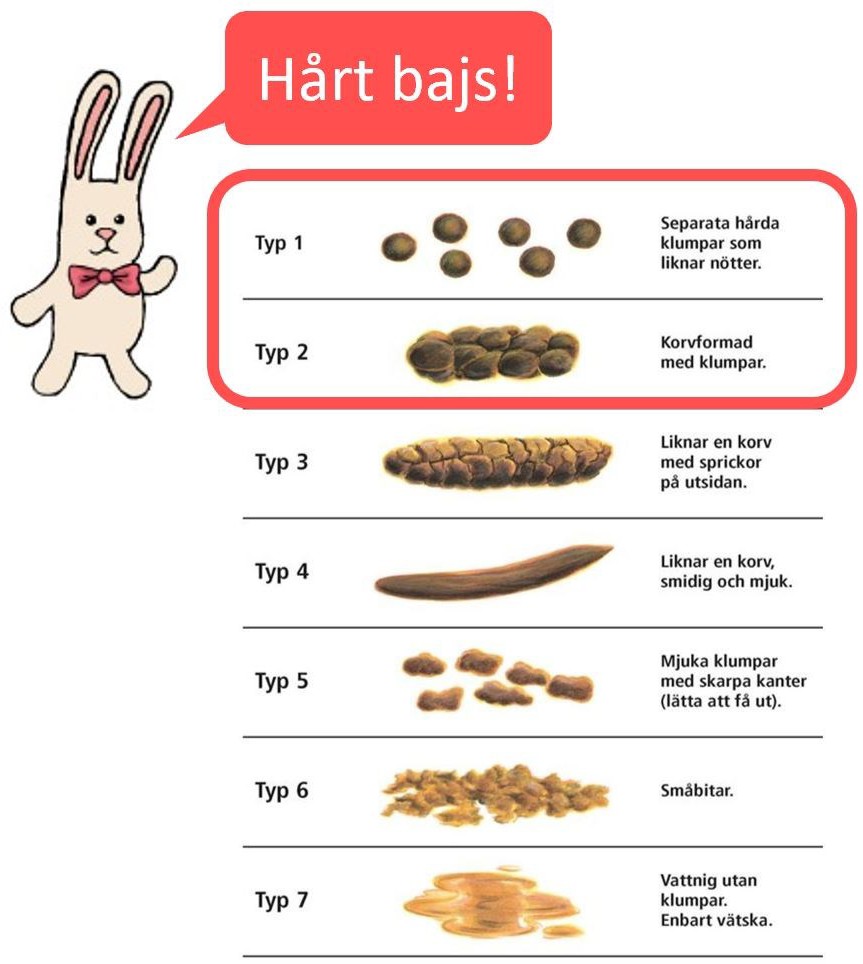


Hard poops!

1. Did your child go through a phase of consistently avoiding using the potty/toilet for bowel movements (despite being comfortable peeing there), asking instead for a diaper to poop in?
   1. Yes, for more than 1 month
   2. Yes, for less than 1 month
   3. No
2. How old was your child when this occurred? *Several options can be chosen*
   1. 0-1 year (0-11 months)
   2. 1-2 years (12-23 months)
   3. 2-3 years (24-35 months)
   4. 3-4 years (36-47 months)
   5. Ongoing

Comments (optional):______________________________________________________

*IN THE LAST MONTH:*

1. Does your child attend preschool/daycare?
   1. Yes
   2. No
   3. Other
2. How many hours a week does your child spend in preschool/daycare, on average?
   1. 20 hours a week or less
   2. 21-39 hours a week
   3. 40 hours a week or more
3. Does your child use the potty/toilet to pee at preschool/daycare?
   1. Yes, often
   2. Yes, sometimes
   3. No, my child always pees in a diaper
   4. No, my child refrains from peeing
4. Does your child use the potty/toilet to poop at preschool/daycare?
   1. Yes, often
   2. Yes, sometimes
   3. No, my child always poops in a diaper
   4. No, my child refrains from having poops

How accurate are the following statements?

*Mark the figure describing the situation*

1. My child received sufficient support in toilet training from preschool/daycare?

Completely disagree Completely agree

1 2 3 4 5 6

1. Preschool/daycare accommodates regular toilet routines for my child.

Completely disagree Completely agree

1 2 3 4 5 6

1. Please describe your experiences and reflections on toilet training and toilet routines at preschool/daycare. Consider both positive and negative aspects.

______________________________________________________________________________________________________________________________________________________________

*SINCE THE LAST SURVEY:*

1. Have there been major changes in your child’s daily life?
   1. No
   2. Yes, the arrival of a sibling
   3. Yes, change of residence
   4. Yes, separation
   5. Yes, illness in the family/close relatives
   6. Yes, death in the family/close relatives
   7. Yes, other

Comments (optional):______________________________________________________

1. Are you living with your child’s other parent/legal guardian?
   1. Yes, I am married/cohabiting
   2. I am separated with joint custody of my child.
   3. No, I am separated with sole custody of my child
   4. Other

Comments (optional):______________________________________________________

1. To what extend does your child live with you?
   1. More than 75% of the time
   2. 25-75% of the time
   3. Less than 25% of the time
2. Are there other children (e.g. full /half or step-siblings) in the household?
   1. Yes
   2. No
3. Specify the ages of the other children (e.g. full /half or step-siblings) and indicate whether they live full-time or part-time in the household:

The sibling lives in the household:

Age (years):_____

- >75% of the time
- 25-75% of the time
- >25% of the time

*(Automatic display of as many options as needed in web-survey)*

Comments (optional):______________________________________________________

1. Indicate if any of the child's (biological) parents or siblings (full- or half-siblings) have or have had the following issues:
   1. Constipation (required medical attention/received treatment)
   2. Bedwetting (after 6 years of age)
   3. Neuro-psychiatric impairment (e.g ADHD, autism, Asperger’s)

Mother a b c

□ Yes □ Yes □ Yes

□ No □ No □ No

□ I don’t know □ I don’t know □ I don’t know

□ Not applicable □ Not applicable

Father a b c

□ Yes □ Yes □ Yes

□ No □ No □ No

□ I don’t know □ I don’t know □ I don’t know

□ Not applicable □ Not applicable

Sibling 1 a b c

□ Yes □ Yes □ Yes

□ No □ No □ No

□ I don’t know □ I don’t know □ I don’t know

□ Not applicable □ Not applicable

*Siblings can be specified by filling in the bottom row of the table. This can be repeated to add additional siblings as needed*

Comments (optional):______________________________________________________

1. Has your child’s development and growth been normal (according to assessments during visits to the Child Health Center)?
   1. Yes
   2. No

Comments (optional):______________________________________________________

1. Is your child allergic to cow’s milk protein (confirmed by healthcare professionals)?
   1. Yes
   2. No
   3. No, but my child has been allergic to cow’s milk protein before
2. Is your child gluten intolerant (confirmed by healthcare professionals)?
   1. Yes
   2. No
3. Has your child suffered from other stomach issues (not including constipation) that prompted you to seek medical care at any time?
   1. Yes
   2. No

Comments (optional):______________________________________________________

1. Did your child ever receive treatment with antibiotics for urinary tract infection?
   1. Yes
   2. No
2. Does your child regularly take any prescribed medications?
   1. Yes
   2. No
3. What prescribed medications does you child take?

_______________________________________________________________________

1. Does your child suffer from any health condition or malformation affecting the bowel or urinary tract?
   1. Yes
   2. No
   3. I don’t know

Comments (optional):______________________________________________________

1. Have you or anyone close to your child suspected neuro-psychiatric impairments in your child?
   1. Yes
   2. No
   3. I don’t know

Comments (optional):______________________________________________________
