# Supplementary material for: The BABITT questionnaire for evaluation of bowel and bladder function in children who are introduced to assisted infant toilet training - content validity and feasibility
Source: PLoS One. 2025 Apr 30;20(4):e0320564. doi: 10.1371/journal.pone.0320564 (PMC12043185; doi:10.1371/journal.pone.0320564)
Supplement: Attachment — Submitted filename: Reviewer Comments .docx [file pone.0320564.s005.docx]

I had the privilege to review a paper titled The BABITT questionnaire for evaluation of bowel and bladder function in children who are introduced to assisted infant toilet training - content validity and feasibility. This is a very interesting paper, however I have a few comments which need to be clarified.

**Study design**

The study has not stated what design was used. The authors have just described how the questionnaire was structured and the recruitment process. The first thing that should have been mentioned in the first sentence is the study design.

**Results**

Check table 2, not all results are showing. Consider using the landscape since this is a wide table.

This also applies to tables 3 and 4.

Conclusion

The conclusion is very brief and has not highlighted the findings of the study. This section must tie the findings together, giving an overall conclusion.

Overall, there are so many limitations, including failure to access the papers that are fundamental to this study, lack of qualitative results except focus group discussions. With all these critical limitations what makes this study to be reliable in terms of internal and external validity?
